# Unlocking the Antioxidant, Enzyme Inhibitory and Acaricidal Potential of *Azadirachta indica* Phytoconstituents Using In Vitro and In Silico Approaches

**DOI:** 10.1002/fsn3.71204

**Published:** 2025-11-29

**Authors:** Tehreem Fatima, Mazhar Abbas, Kinza Zafar, Maha Gul Zafar, Waqas Haider, Muhammad Haseeb Zafar, Muhammad Riaz, Munawar Iqbal, Andrew G. Mtewa

**Affiliations:** ^1^ Department of Basic Sciences (Section Biochemistry) University of Veterinary and Animal Sciences Lahore, Jhang Pakistan; ^2^ Department of Gynecology and Obstetrics Railways Hospital, Islamic International Medical College Rawalpindi Pakistan; ^3^ Medical Unit 3 Lahore General Hospital Lahore Pakistan; ^4^ Department of Allied Health Sciences University of Sargodha Sargodha Pakistan; ^5^ School of Chemistry University of the Punjab Lahore Pakistan; ^6^ Renewable Energy and Environmental Technology Center Saudi Arabia University of Tabuk Tabuk Saudi Arabia; ^7^ Chemistry Section, Malawi Institute of Technology Malawi University of Science and Technology Limbe Malawi

**Keywords:** acaricidal, cytotoxic, glutathione transferase, neuroactive, Ramchandran plot, *S. scabiei*

## Abstract

The study evaluated the acaricidal potential of bioactive components of *Azadirachta indica* against scabies mortality using both in vitro and in silico approaches. *Sarcoptes scabiei* were stimulated with *A. indica* at four concentrations (25–100 mg/mL) at different intervals. The study assessed the cytotoxic, neuroactive, and detoxification‐modulating potential of *A. indica* , emphasizing their antibacterial, antioxidant, and enzyme‐inhibitory potential. LCMS was used for the characterization of phytoconstituents. In silico analysis encompassed target prediction, toxicity assessment, biological activity prediction, protein structure modeling, and gene expression analysis. Molecular docking assesses the binding affinities of bioactive components, and the ExPASy database predicts the physiochemical properties of glutathione transferase. In vitro analysis suggests that *A. indica* has a dose‐dependent effect on *S. scabiei* at different time intervals. It highlights the extract's multifaceted bioactivity with strong antioxidant activity (IC_50_ = 3.15 mg/mL) and potent antibacterial effects at higher concentrations. It exhibited mild to moderate hemolytic and significant AChE activity. Furthermore, it also showed GST inhibition, suggesting possible disruption of toxins. The binding affinity of 7‐desacetyl‐7‐benzoylazadiradione showed significant inhibitory interaction with glutathione transferase (−12.324 kcal/mol). This phytoconstituent exhibited a high hyper‐geometric *p* value; the total interactions between transcription factors, kinases, and intermediate proteins are observed. *A. indica* serves as a natural substitute for managing mite infestations and offering insights into mechanisms by which its phytochemicals show inhibitory effects.

## Graphical abstract

The study evaluated the acaricidal potential of bioactive components of *Azadirachta indica* against scabies mortality using both in vitro and in silico methodologies. *Sarcoptes scabiei* were stimulated with *Azadirachta indica* at four concentrations (25–100 mg/mL) at different intervals. The study assessed the cytotoxic, neuroactive, and detoxification‐modulating potential of *A. indica*, emphasizing their antibacterial, antioxidant, and enzyme‐inhibitory potential. LCMS was used for the characterization of phytoconstituents.

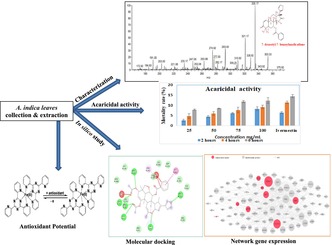

**Abbreviations**
ABL1: abelson tyrosine kinase 1
ADME: absorption, distribution, metabolism, and excretion
AKT1: serine–threonine protein kinase
ARG: 58: arginine 58
ARG: 107: arginine 107
BBB: blood brain barrier
BLAST: basic local alignment search tool
BS: biostability
CNR1: cannabinoid receptor 1
CNR2: cannabinoid receptor 2
CSNK2A1: casein kinase 2 alpha 1
CTSK: cathepsin K
CTSV: cathepsin V

*E. coli*

:
*Escherichia coli*

ESOL Log‐S: water solubility
ExPASy: expert protein analysis system
FASN: fatty acid synthase
GIA: gastrointestinal tract
GRAVY value: grand average of hydropathicity
GSK3B: glycogen synthase kinase 3 beta
GST: glutathione transferase
HDAC1: histone deacetylase 1
HHblits: HH‐suite‐based iterative search
IKKBETA: inhibitor of nuclear factor kappa‐B kinase subunit beta
KCNA5: potassium voltage‐gated channel subfamily A member 5
LCMS: liquid chromatography–mass spectrometry
LEU: 57: leucine 57
Log‐Kp: skin permeation
Log‐Po/w: lipophilicity consensus log
LYS: 54: lysine 54
MAP K11: mitogen‐activated protein kinase 11
MAP K14: mitogen‐activated protein kinase 14
MW: molecular weight
NIST: National Institute of Standards and Technology
PDB: protein data bank
PFKFB3: 6‐phosphofructo‐2‐kinase/fructose‐2,6‐biphosphatase 3
P‐gp: P‐glycoprotein
RT: retention time

*S. scabiei*

: *Sarcopetes scabie*
SER: 56: serine 56
SMTL: SWISS‐MODEL template library
SRT: silent information regulator 2‐related enzymes
SsGST: *Sarcopetes scabiei* glutathione transferase
SUZ1: polycomb repressive complex 2 subunit
THR: 59: threonine 59
TNKS2: tankyrase 2
TPSA: topological polar surface area
trpA: tryptophan A
trpB: tryptophan B
trpD: tryptophan D
UBTF: upstream binding transcription factor
VADAR: volume area dihedral angle reporter
WNT3A: Wnt family member 3A
X2K: eXpression2Kinases

## 1 Introduction

The astigmatic ectoparasite *Sarcoptes scabiei* from the subfamily Sarcoptinae causes scabies in humans and spreads indirectly or directly between humans and animals. Humans become temporarily infected with zoonotic strains of *S. scabiei* through contact with infected animals, causing self‐limiting illness, pseudo‐scabies. In scabies outbreaks, person‐to‐person transmission is more significant and persistent (Nardoni and Mancianti 2022). These mites invade the stratum corneum of the epidermis, resulting in distinctive cutaneous symptoms. More than 150 host species are infected by *S. scabiei* globally, demonstrating its versatility to spread among a wide range of hosts (Moroni et al. 2022).

Sarcoptic mange spreads through the exposure of contaminated surfaces to areas with infested hosts and direct skin contact (Bernigaud et al. 2020). *S. scabiei* adult females and nymphs can survive longer than those of larval males up to 21 days. The biocides and repellents remove them from the environment and halt their spread (Fang et al. 2015). The high mortality and morbidity rates of *S. scabiei* in both domestic and wild mammals result in substantial economic losses. The skin becomes exposed to secondary bacterial infections, such as those caused by *Staphylococcus aureus* and *Streptococcus pyogenes* (Andriantsoanirina et al. 2022). The symptoms of scabies in animals vary from mild to chronic conditions, including itchy papules, redness, scales, and hair loss. It leads to crusting, skin thickening, and hyperkeratosis (Cardells et al. 2021). While scabies result in weight loss, decreased fiber production and quality in rabbits, and cause conditions like dermatitis, urticaria, eczema, and pyoderma (Khan et al. 2022).

The synthetic acaricides, including macrocyclic lactones, phenylpyrazoles, synthetic pyrethroids, formamidines, organophosphates, and growth inhibitors, disrupt the life cycle of mange mites by interfering with ligand‐gated channels and eliminating infestations (Fantatto et al. 2022). The usage of acaricides has caused tick resistance, which has raised interest in economical plant‐based alternatives (Eid et al. 2017). *A. indica* (neem) offers a possible substitute for synthetic medication attributed to a wide range of its effectiveness, low toxicity, and ability to strengthen the immune system, as well as bioactive natural products for treating parasitic diseases, and especially *S. scabiei* (Gupta et al. 2019). Acquiring bioactive phytocompounds and their effects on *S. scabiei* is necessary to clarify the potential defense against scabies, highlighting the need for mechanistic research and innovative therapeutic approaches (Nicoletti 2020). Primarily, the phytochemicals such as 7‐desacetyl‐7‐benzoylazadiradione, azadirachtins, caryophyllene, salannin, nimbin, and 6‐desacetylnimbin, are versatile pesticides, larvicides, and acaricides.

Bioactive compounds demonstrate anti‐inflammatory, antipyretic, antibacterial, anticancer, and immunomodulatory properties. According to (Kotnala et al. 2019), the neem tree's leaves, seeds, bark, and oil display exceptional biological activity. The leaves contain quercetin, catechins, carotenes, effectively fight against scabies, and the leaf extract demonstrates potent antiseptic properties. Extracts from neem seeds counteract veterinary and medical pests like lice, mites, and ticks. Neem oil interrupts mite life cycles by destroying eggs, changing the physiology, and affecting the nervous system (Salehzadeh 2002).

It addresses toxicity, adulteration, species identification, and removing contaminants like pesticide residues and heavy metals (Jităreanu et al. 2022). In silico approaches are used for medicinal ligand and receptor complexes (Haghighi et al. 2020; Rahmati et al. 2020). In ticks, GST helps with toxin neutralization against oxidative damage (Vudriko et al. 2018).

This article forms the foundation for the bioassays to examine the efficacy of each phytoconstituent. We evaluated the mortality rate of *S. scabiei* , antioxidant, antibacterial, hemolytic assay, and enzyme inhibition activities. These analyses shed light on the cytotoxic, neuroactive, and detoxification‐modulating characteristics of plant extracts, leading to the development of new drugs and screening for toxicity in vitro, and ascertaining their insecticidal efficacy against scabies. LCMS identifies the *A. indica* key phytochemicals. Furthermore, it employs in silico analysis such as absorption, distribution, metabolism, and excretion (ADME) pharmacokinetics, biological activity prediction, homology modeling, molecular docking, and analysis of target gene expression.

## 2 Materials and Methods

### 2.1 Plant Collection and Extraction

The leaves of neem ( *A. indica* ) were obtained from the Chenab Park area in the locality of Jhang, Pakistan. These leaves were washed and shade‐dried for 15 days. Leaf powder (100 g) was dissolved in 1 L of 80% ethanol at 300 rpm for 48 h. The agitated solution was condensed at 40°C using a rotary evaporator. The solution was concentrated in a 45°C water bath following ethanol removal. The sample was stored at refrigerated temperature for further analysis (Hakami et al. 2023).

### 2.2 Total Antioxidant Activity

The antioxidant activity of the plant extracts was evaluated using total phenolic content (TPC), total flavonoid content (TFC), and DPPH radical scavenging assay. TPC was determined using the Folin–Ciocalteu method (Sánchez‐Rangel et al. 2013), where the sample was mixed with Folin reagent, incubated, treated with Na_2_CO_3_, and measured at 765 nm. Results were expressed as milligram gallic acid equivalent per gram dry weight (mg GAE/g) of extract. TFC was measured using the aluminum chloride method (Begum and Khan 2025), where the extract was mixed with 0.2 mL of 5% sodium nitrite, 2 mL of 1 M sodium hydroxide and 0.2 mL of 10% aluminum chloride, and absorbance was recorded at 510 nm. The results were expressed as milligram catechin equivalents per gram (mg QE/g). DPPH radical scavenging activity was assessed by mixing the sample with 0.1 mM methanolic DPPH solution followed by incubation in the dark for 40 min, and measuring absorbance at 517 nm. The percentage inhibition was calculated using the formula:

$$\%\text{inhibition of DPPH free radical scavenging activity}=A_{0}-A/A_{0}\times 100$$

where *A*_0_ is the control absorbance and *A* is the sample absorbance. All experiments were conducted in triplicate, and results were expressed as mean ± SD (Goyal et al. 2010).

### 2.3 Antibacterial Assay

A pure culture of bacterial strain was grown in nutrient agar medium. A 1.2 × 10^8^ CFU/mL inoculum of microbe was used. Each sterilized disposable petri plate contained 100 μL of fresh bacterial culture and 20 mL of nutrient agar. The plates were left to solidify for 20 min at room temperature and the wells were made through a borer. Then 100 μL of sample and control (ciprofloxacin 1 mg/mL) were poured in each well. After 10 min, petri plates were placed in an incubator set at 37°C for 24 h. The zone of inhibition was recorded and measured in mm (Altayb et al. 2022).

### 2.4 Hemolytic Assay

The in vitro cytotoxicity analysis of *A. indica* was carried out through a hemolytic assay. For this analysis, a bovine blood sample was collected in a heparinized tube, washed with PBS (pH 7.4), and centrifuged at 850 *g* for 5 min. RBCs were counted after washing three times with PBS and adjusted to 7.068 × 10^8^ cells/mL. Then 50 μL of the stock solutions were treated with 180 μL of diluted blood cell suspension. The sample mixture was incubated at 37°C for 30 min. Then, the sample was agitated and centrifuged for 5 min at 1310 *g*. After centrifugation, 100 μL supernatant samples were diluted with 900 μL chilled PBS, transferred to a 96‐well plate, and analyzed at 576 nm using a BioTek μQuant microplate reader. The PBS (0% lysis) and 0.1% Triton X‐100 (100% lysis) were used as controls (Abbas et al. 2018).

### 2.5 Enzyme Inhibition Assays

GST inhibition was assessed using mono chlorobimane (MCB) and 1‐Chloro‐2,4‐dinitrobenzene (CDNB) as substrates. In these enzyme inhibition assays, mite homogenates were used as the enzyme source. To the mite homogenate, 200 μL of substrate (3 mg reduced glutathione dissolved in 10 mL of 0.05 M Tris–HCl buffer pH 7.5 + 500 μL of 1.5 mg/mL of MCB in methanol) was added followed by incubating the plant extracts with GST enzyme and mite homogenate for 20 min at room temperature. AChE inhibition was evaluated using the reaction mixture containing 200 μL of AChE enzyme, 1000 μL DTNB reagent, and 200 μL of test compound (25, 50, 75, and 100 mg/mL) incubated at 25°C for 15 min. The reaction was initiated with 200 μL of acetylthiocholine, and inhibition was measured at 412 nm, with galanthamine as the positive control (Hu et al. 2015).

### 2.6 Acaricidal Activity of *A. indica* on Sarcoptic Mange

The 2 mL of diluted extract at 25, 50, 75, and 100 mg/mL containing mites were placed in petri plates and incubated. The reaction was observed for 2, 4, 6 h after application. A positive control group (containing 1% ivermectin) was cultured in a petri plate at 25°C and 75% relative humidity. Mites were confirmed dead by needle stimulation (Zahran et al. 2022).

### 2.7 LCMS Profiling of *A. indica* Extract

For this analysis, 10 mL of LCMS‐grade methanol was used to sonicate 50 mg of neem leaf powder for 30 min. After centrifuging the extract, a PTFE 0.2 μm filter was used to filter the supernatant. To get a clear solution, 50 μL of hydrocortisone (1 μg/mL) was added to 450 μL of neem extract and vortexed (Biswal et al. 2021). LCMS detected the *A. indica* phytoconstituents from prepared samples using a multichannel solid phase extraction (SPE) cartridge with Strata C18 columns and a vacuum pump (Sun et al. 2007). An Agilent 1290 Infinity LC system with ESI and Q‐TOF 6520 mass spectrometer was used for LCMS analysis. It employed the Zorbax Eclipse XDB‐C18 column. LCMS measured the phytoconstituent's retention time, mass‐to‐charge ratio, and relative intensity (Rahim et al. 2022).

### 2.8 In Silico Study

#### 2.8.1 In Silico Pharmacokinetics

SMILES of each ligand were used to screen for ADME using the Swiss ADME server (http://www.swissadme.ch) (Ranjith and Ravikumar 2019).

#### 2.8.2 In Silico Target Prediction

The Swiss Target Prediction system (http://www.swisstargetprediction.ch/) identified *Homo sapiens* as the target organism and *A. indica* chosen ligands with a high skin‐permeability coefficient according to the pharmacokinetics (Daina et al. 2019).

#### 2.8.3 Biological Activity Prediction

Data Warrior 5.0.0 was utilized to study phytochemical toxicology, predicting mutagenic, tumorigenic, reproductively effective, and irritating effects. AdmetSAR‐2.0 (http://lmmd.ecust.edu.cn/) was used for metabolism, biodegradation, in silico toxicity of rat and fish models and fatal dosage assessment (Asha et al. 2021).

#### 2.8.4 Homology Modeling

The study used homology modeling to predict the 3D structure of a pharmacological target, *Sarcoptes scabiei* glutathione transferase, using UniProt accession No: Q8I9R9. The SWISS‐MODEL protein modeling server was used to predict the three‐dimensional structure of the SsGST protein, using BLASTp and HHBlits tools for target template sequence alignment, referencing the Protein Data Bank (PDB) and SWISS‐MODEL Template Library (SMTL) repositories. After homology modeling, the structure was downloaded in PDB format (Hakami et al. 2023). The physicochemical properties of glutathione transferase were predicted using the ProtParam program from the ExPASy database (https://web.expasy.org/protparam/) (Kaur et al. 2020).

#### 2.8.5 Ligand Preparation

LCMS‐validated phytochemicals' SMILES structures used from NCBI PubChem Compound database (https://pubchem.ncbi.nlm.nih.gov/). Structures were drawn by Chem Sketch and 3D made with Discovery Studio.

#### 2.8.6 Analysis of Molecular Docking

Homology modeling and PDB generated the target protein's 3D structure. High‐skin‐penetration ligands were optimized in 3D and saved in Mol format using ACDLab/Chemsketch 2‐dimensional ligand‐protein interaction by Discovery Studio (Ahammad et al. 2021).

#### 2.8.7 Analysis of Target Gene Expression

For this analysis, highly bound target proteins—HDAC1, CNR1, CNR2, KCNA5, PFKFB3, MAPK14, CTSK, CTSV, MAPK11, FASN, WNT3A, GSK3B, AKT1, ABL1, IKKBETA, CSNK2A1, and TNKS2—were utilized. These gene IDs were gathered and utilized using the eXpression2Kinases (X2K) Web server https://maayanlab.cloud/X2K/ for expression network analyses (transcription factor enrichment analysis, protein–protein interaction network expansion, and kinase enrichment analysis) (Fatoki et al. 2022).

#### 2.8.8 Statistical Analysis

All the values were taken in triplicate to reduce bias and represented as mean ± standard deviation. Using software (SPSS Statistics v22.0, IBM), the percentage mortality values and 95% confidence limits were computed. Using SPSS Statistics v22.0 (IBM), a one‐way analysis of variance (ANOVA) was used to analyze the data and to identify the differences among the experimental groups. Results with a *p* value < 0.05 were considered statistically significant.

## 3 Results and Discussion

### 3.1 Total Antioxidant Activity

The antioxidant potential of neem plant extract was evaluated through Total Phenolic Content (TPC), TFC, and DPPH radical scavenging activity, all demonstrating a dose‐dependent increase. TPC values increased with increasing concentration, from 15.68 ± 0.54 mg GAE/g DW at 25 mg/mL to a maximum of 20.82 ± 0.94 mg GAE/g DW at 100 mg/mL, indicating a substantial presence of phenolic compounds at higher doses. Similarly, TFC followed a concentration‐dependent rise, from 9.31 ± 0.44 mg QE/g DW at 25 mg/mL to 13.71 ± 0.78 mg QE/g DW at 100 mg/mL. The DPPH assay confirmed the enhanced free radical scavenging activity, with inhibition increasing from 20.56% ± 1.13% at 25 mg/mL to 4.28% ± 1.62% at 100 mg/mL and the control (Ascorbic acid) value at 100 μg/mL is 85.88% ± 1.01% as shown in Table 1. The IC_50_ value of DPPH at 3.15 mg/mL highlights strong antioxidant potency, demonstrating the neem extract's effectiveness in neutralizing free radicals. These results are consistent with previous research on *Azadirachta indica. A. indica* is widely known as a natural biopesticide and showed antioxidant potential that varies across different extracts, with the highest activity in butanol extract (Al‐Hashemi and Hossain 2016; Septiyani and Wibowo 2019).

**TABLE 1:** TPC, TFC, and DPPH radical scavenging activities of *Azadirachta indica* .

Concentration	TPC (mg GAE/g DW)	TFC (mg QE/g DW)	DPPH radical scavenging activity
*A. indica* (25 mg/mL)	15.68% ± 0.54%	9.31% ± 0.44%	20.56% ± 1.13%
*A. indica* (50 mg/mL)	17.03% ± 0.69%	10.95% ± 0.51%	35.55% ± 1.34%
*A. indica* (75 mg/mL)	19.40% ± 0.75%	11.65% ± 0.59%	43.33% ± 1.58%
*A. indica* (100 mg/mL)	20.82% ± 0.94%	13.71% ± 0.78%	64.28% ± 1.62%
Control (100 μg/mL ascorbic acid)	—	—	85.88% ± 1.01%

Various parts of neem have been studied for potential antioxidant activity, including neem leaf flour and root, indicating the presence of key phenolic and flavonoid compounds. These protect the cells and tissues from oxidative damage and possess anticancer, anti‐inflammation, anti‐diabetes, neuroprotective, and antimutagenic potential (Andersa et al. 2024; Hussain et al. 2023; Kaur et al. 2024).

### 3.2 Antibacterial Activity

The extract (25 mg/mL) exhibited antibacterial activity with inhibition zones against *B. subtilis*, *E. coli*, *C. perfringens* and *S. equi* as 13.00 ± 0.81 mm, 12.00 ± 0.81 mm, 11.3 ± 0.6 mm and 12.7 ± 0.6 mm while other concentrations showed various degrees of effectiveness (Table 2). The findings show enhanced antibacterial effectiveness at increasing doses. The greatest antibacterial activity was shown by the largest inhibition zone, seen at the highest tested concentration (100 mg/mL). The plant extracts of *Azadirachta indica, Eucalyptus globulus, Millettia ferruginea*, and *Euphorbia abyssinica* have reportedly acaricidal activity against these mites (Mohammed 2019). Research by (Abd El‐Moez et al. 2014), demonstrated that neem extract efficiently inhibited *B. subtilis* , *E. coli* , *C. perfringens* , and *S. equi* with inhibition zones ranging from 10 to 17 mm. The results confirm its function in disrupting bacterial cell walls and inhibiting their growth.

**TABLE 2:** Antibacterial efficacy of *Azadirachta indica* against Gram‐positive and Gram‐negative bacterial strains.

Concentrations	*Bacillus subtilis* (ATCC 6633), mm	*Escherichia coli* (ATCC 8730), mm	*Clostridium perfringens* (ATCC 13124), mm	*Streptococcus equinus* (ATCC 9528), mm
*A. indica* (25 mg/mL)	13 ± 0.81	12 ± 0.81	11.3 ± 0.6	12.7 ± 0.6
*A. indica* (50 mg/mL)	14.25 ± 0.95	14.5 ± 1.29	13 ± 0.7	13.3 ± 0.8
*A. indica* (75 mg/mL)	15.5 ± 1.29	15.75 ± 1.5	15 ± 0.8	14.5 ± 0.9
*A. indica* (100 mg/mL)	16.75 ± 1.5	17 ± 1.82	17.7 ± 1.0	15 ± 0.92
Ciprofloxacin (10 mg/mL)	35.98 ± 1.1	38.62 ± 1.0	38.84 ± 1.2	35.01 ± 1.1

*Note:* The zone of inhibition measured in mm and the values are the mean of triplicate measurements (*n* = 3) ± SD.

### 3.3 Cytotoxicity

The cytotoxic potential of *A. indica* was measured through a hemolytic assay using bovine red blood cells for screening safer bioactive compounds that are viable for drug formulations (Ghazali et al. 2022). The results of the hemolysis assay revealed that RBC lysis increased in a concentration‐dependent manner with hemolysis rates of 5.07% ± 1.08% at 25 mg/mL, 6.27% ± 1.97% at 50 mg/mL, 7.77% ± 1.25% at 75 mg/mL, and 8.50% ± 1.65% at 100 mg/mL. Assay reliability was confirmed when the positive control (Triton X‐100) exhibited 98.37% ± 2.9% hemolysis. This assay validates that this safe and efficient plant‐based anti‐scabies medication, does not significantly damage red blood cells, aiding in the selection of an effective treatment drug.

### 3.4 Enzyme Inhibition

Glutathione S‐transferases are the key biomarkers of oxidative stress and play an important role in the detoxification of reactive electrophiles and toxins. These antioxidant enzymes protect DNA and protein from oxidative damage (Abu‐Elala et al. 2023; Nikolova et al. 2022). The GST inhibitory effect of *A. indica* showed 40.35% ± 1.59% and 56.99% ± 1.98% inhibition at 25 and 100 mg/mL, respectively. The control PBS has 76.12% ± 0.97% inhibition at 100 μg/mL, suggesting potential GST inhibition. Results demonstrate a significant concentration‐dependent increase while exhibiting lower activity than control, strongly reinforcing its therapeutic potential as shown in Table 3. In contrast, the AChE assay showed a dose‐dependent increase in enzyme activity, with 32.67% ± 1.17% inhibition at 25 mg/mL, rising progressively to 78.49% ± 1.41% at 100 mg/mL. The Galanthamine showed 77.90% ± 0.92% inhibition at 100 μg/mL, indicating the sample serves as a potential AChE inhibitor. These results exhibit that *A. indica* possesses neurotoxic activity on insects by inhibiting the neural transporters tyramine and acetylcholinesterase‐dependent γ‐aminobutyric acid (GABA) sodium and chloride channels (Khallaf et al. 2024).

**TABLE 3:** GST and AChE inhibitory activity of *Azadirachta indica* .

Concentrations	% GST inhibition	% AChE inhibition
*A. indica* (25 mg/mL)	40.35 ± 1.59	32.675 ± 1.17
*A. indica* (50 mg/mL)	41.72 ± 1.77	45.86 ± 1.31
*A. indica* (75 mg/mL)	48.75 ± 1.81	61.875 ± 1.36
*A. indica* (100 mg/mL)	56.99 ± 1.98	78.495 ± 1.41
Control at (100 μg/mL)		
PBS	76.12 ± 0.97	—
Galanthamine	—	77.90 ± 0.92

### 3.5 Acaricidal Activity of *A. indica* on Sarcoptic Mange

**FIGURE 1:**
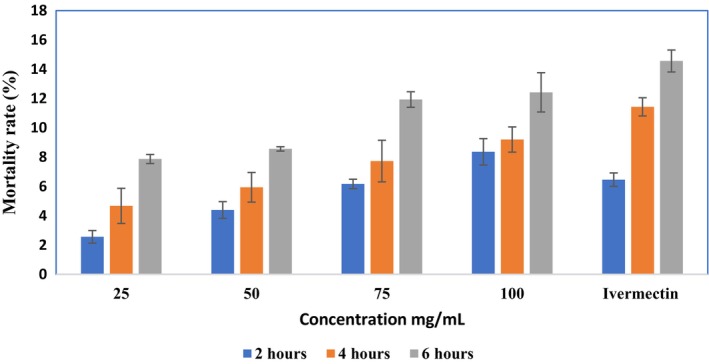
Determination of lethal time with respective concentrations of *A. indica*.

This assay evaluates the efficacy of *A. indica* at 2‐, 4‐, and 6‐h intervals post‐application. For instance, at the 2‐h mark, the mean values for concentrations of 25, 50, 75, and 100 mg/mL were 2.56% ± 0.43%, 4.39% ± 0.57%, 6.17% ± 0.32%, and 8.36% ± 0.90%, respectively. At the 6‐h mark, the mean values reached 7.87% ± 0.31%, 8.56% ± 0.15%, 11.93% ± 0.53%, and 12.42% ± 1.34% for concentrations of 25, 50, 75, and 100 mg/mL, respectively (Table 4, Figure 1). The results showed statistically significant differences among research groups. A *p* value of 0.01 typically shows a significant difference from the other groups (File S1). Findings suggest that certain phytoconstituents in neem potentially counteract the commercially used acaricide permethrin's lower toxicity. According to reports, a topical spray containing 25% aqueous neem fruit extract was exceptionally effective in treating mange mites as commercial amitraz medications, and the treated pigs experienced no negative side effects (Khan et al. 2022; Pasipanodya et al. 2021).

**TABLE 4:** The death rates of Sarcoptes *scabiei* by *Azadirachta indica* extract in vitro.

	Concentration	2 h	4 h	6 h
*Azadirachta indica* (mortality rate %)	25 mg/mL	2.56% ± 0.43%	4.67% ± 1.2%	7.87% ± 0.31%
	50 mg/mL	4.39% ± 0.57%	5.94% ± 1.01%	8.56% ± 0.15%
	75 mg/mL	6.17% ± 0.32%	7.73% ± 1.42%	11.93% ± 0.53%
	100 mg/mL	8.36% ± 0.90%	9.20% ± 0.86%	12.42% ± 1.34%
Positive control	Ivermectin 1% w/v	6.464% ± 0.467%	11.431% ± 0.623%	14.56% ± 0.75%

### 3.6 LCMS Profiling of *A. indica* Extract

**FIGURE 2:**
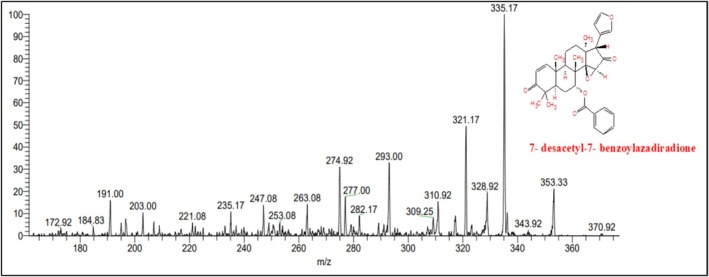
LCMS chromatogram of *A. indica* extract.

The characterization of ethanol extracts of *A. indica* by LCMS/MS is summarized in Table 5. Peaks were identified by comparing with published libraries (NIST and European mass data bank), referring to retention times, fragmentation patterns, and molecular weight estimates (Figure 2). Additional data from the Phenol‐Explorer database was also used to authenticate peaks.

**TABLE 5:** Identified compounds in *A. indica* extract.

Sr. #	Compound	*m*/*z* M (+/−)	RT	Medical importance
1.	Azadirachtin A (azadirachtin) 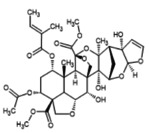	−719.6	2.15	It possess anti‐cancerous, osteogenic, and antioxidant potential (Islas et al. 2020; Nwanekezie et al. 2023)
2.	Azadirachtin‐B (3‐tigloylazadirachtol) 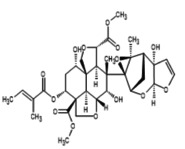	663.6	2.2	It has role in Increased proliferation, differentiation and mineralization in osteoblasts (Islas et al. 2020)
3.	Azadirachtin D(1‐tigloyl‐3‐acetyl‐11‐hydroxy‐meliacarpin) 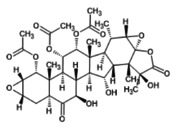	677.7	2.32	It exhibits anticancer and antiproliferative‐activity (Nagini et al. 2024)
4.	Azadirachtin‐H(11‐demethoxycarbonyl azadirachtin) 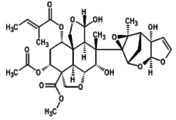	−675.7	2.4	It demonstrates insect antifeedant and has ecdysis‐inhibiting activity (Kharwar et al. 2020)
5.	Azadirachtin I(1‐tigloyl‐3acetyl‐11‐hydroxy‐11‐demethoxycarbonyl meliacarpin) 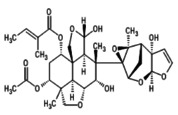	619.7	2.56	It has antifeedant activity and possess cytotoxic effects. It also acts as biopesticides (Kilani‐Morakchi et al. 2021; Nagini et al. 2024)
6.	Azadiradione 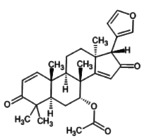	451.6	2.76	It is key plant metabolite, acts as antimycobacterial and anti‐inflammatory agents (Jana et al. 2024; Srivastava et al. 2020)
7.	Deacetyl‐nimbin 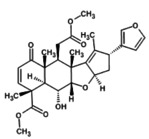	499.5	2.94	It shows anti‐inflammatory, antiangiogenic and antitumor activity (Sudhakaran et al. 2022; Yadav et al. 2016)
8.	Epoxyazadiradione 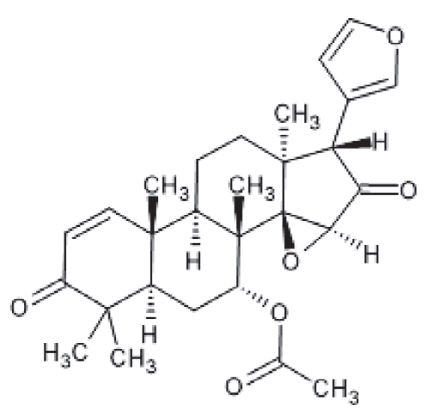	−465.5	3.02	It has anti‐plasmodial, anti‐inflammatory and cytotoxic activities. It suppresses breast tumor growth (Islas et al. 2020; Kumar et al. 2018; Yadav et al. 2017)
9.	Nimbin 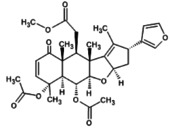	541.6	3.33	It exhibits fungicidal, anti‐inflammatory, antihistamine, antipyretic, and antiseptic effects (Maji and Modak 2021)
10.	Nimbolin A 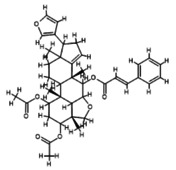	−643.7	3.58	It possesses antioxidant, anti‐inflammatory, antiseptic, and anthelmintic (Hussain et al. 2023)
11.	Nimbandiol 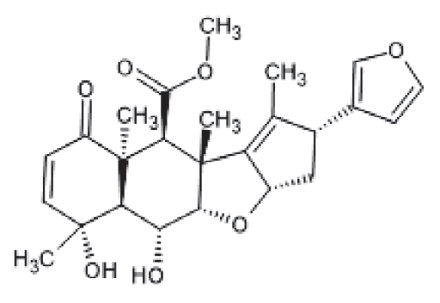	443.5	3.58	It has anti‐inflammatory, cytotoxic, and antimycobacterial potential (Srivastava et al. 2020)
12.	Nimocinol 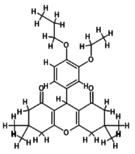	453.6	3.66	It has antioxidant, anti‐inflammatory, anticancer, antifungal, hepatoprotective and wound healing activities (Gupta et al. 2019)
13.	Nimbinene 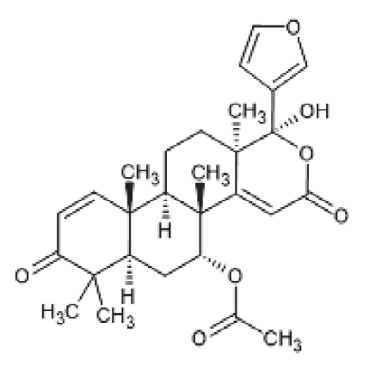	482.5	3.76	It exhibits anti‐inflammatory, cytotoxic, and antimycobacterial activities (Srivastava et al. 2020)
14.	Nimbocinone 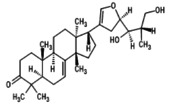	471.6	3.88	It is non‐mutagenic, non‐hepatotoxic, and non‐cytotoxic. It possesses antibacterial and antioxidant activity (Hussain et al. 2023; Raju et al. 2023)
15.	Nimbocinolide 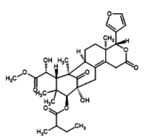	501.5	3.96	It is used in the treatment of lung cancer and it inhibits proliferation, and invasion of cancerous cells (Islas et al. 2020; Nath et al. 2023; Raju et al. 2023)
16.	Nimbolide 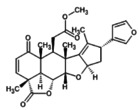	−465.5	4.14	It possesses antioxidant, anti‐malarial, and hepatoprotective (Maji and Modak 2021; Tiwari 2022)
17.	Meliantriol 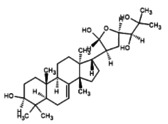	491.7	4.95	It acts as viable food constituents for diabetics and bulking agents (Patel et al. 2024)
18.	Caryophllene 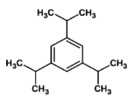	205.3	4.52	It acts as antioxidant and antiaging substance involved in reducing‐melanin synthesis (Wagh et al. 2022)
19.	n‐hexacosanol 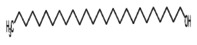	383.7	4.74	It has antinociceptive, anti‐Inflammatory, antibacterial, and antifungal activity (Kabir 2020; Srivastava et al. 2020)
20.	7‐desacetyl‐7‐ benzoylazadiradione 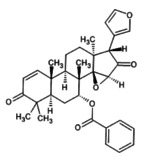	545.6	4.82	It exhibits cytotoxicity‐against leukemia‐cells and have antiproliferative effect (Nagini et al. 2024)
21.	17‐ hydroxyazadiradione 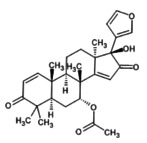	456.5	4.88	It possesses antimicrobial and anti‐inflammatory activity (Borkotoky and Banerjee 2021)
22.	Gedunin 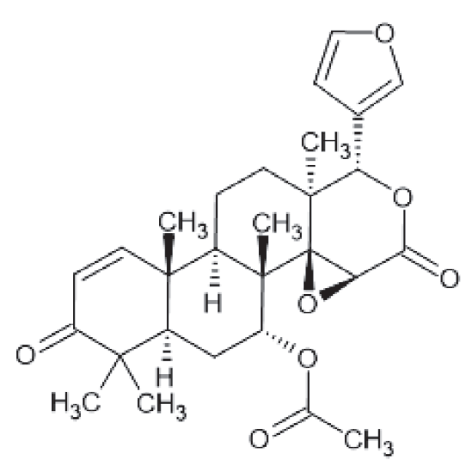	−481.6	4.91	It has antifungal and anti‐ malarial (Maji and Modak 2021)
23.	Vepinin 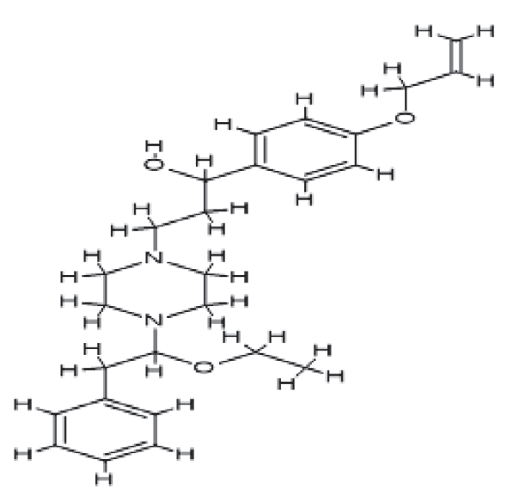	−451.6	4.94	It possesses hypoglycemic activity (Kumar et al. 2014)
24.	Gallic Acid 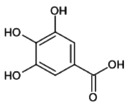	−169.12	4.96	It has anti‐inflammatory, antibacterial, anticancer, Immunomodulatory, antiviral, anti‐cholesterol, and antiulcer activity (Maji and Modak 2021; Zahrani et al. 2020)

Nimbolide, shows an *m*/*z* M of 501.5 and a RT value 3.96, Meliantriol having an *m*/*z* M value of 491.7 and a RT value of 4.95, Caryophyllene shows an *m*/*z* M value of 205.3 and a RT value of 4.52, N‐hexacosanol has an *m*/*z* M value of 383.7 and its RT value is 4.74, and 7‐desacetyl‐7‐benzoylazadiradione shows an *m*/*z* M of 545.6 and a RT value of 4.82. They are natural compounds with anti‐malarial, antiproliferative, and melanogenesis effects, respectively having different molecular ion positions found in neem, and caryophyllene. These results provide insights into the molecular structures and fragmentation patterns of these compounds, aiding in their identification and characterization within the sample analyzed.

### 3.7 ADME Analysis

Swiss ADME developed a reliable and precise approach for interpreting the ADME characteristics of phytoconstituents of *A. indica* extract (Sravika et al. 2021). Several phytochemicals met Lipinski's rule of five, enabling the selection of skin‐permeant compounds for topical scabies treatment. Results of drug‐likeliness of phytoconstituents in Table 6 show that when skin permeation (log kP) was evaluated at a cut‐off value of −6.0 cm/s, eight phytoconstituents of *A. indica* (Azadiradione, Nimbolin A, Nimocinol, Nimbocinone, Meliantriol, Caryophyllene, 7‐desacetyl‐7‐benzoylazadiradione and Vepinin) were found to have dermal penetration activity. Phytochemicals are impermeable to the blood–brain barrier and have poor absorption in the gastrointestinal system showing a 40%–50% likelihood class according to Abbot Bioavailability scores. These findings imply that *A. indica* could potentially develop into paramount nutraceutical and traditional medicinal products.

**TABLE 6:** *A. indica* phytochemicals predicted ADME parameters.

Phytochemicals	MW	TPSA	Log Kp cm/s	ESOL Log S	GIA	BBB	P‐gp	Log Po/w	BS
Azadirachtin A (azadirachtin)	720.71	215.34	−9.92	−4.34	Low	No	Yes	1.08	0.17
Azadirachtin B (3‐tigloylazadirachtol)	662.68	189.04	−9.43	−4.23	Low	No	Yes	1.15	0.17
Azadirachtin D (1‐tigloyl‐3‐acetyl‐11‐hydroxy‐meliacarpin)	676.70	189.04	−8.97	−4.81	Low	No	Yes	1.55	0.17
Azadirachtin H (11‐demethoxycarbonyl azadirachtin)	676.70	189.04	−8.97	−4.81	Low	No	Yes	1.55	0.17
Azadirachtin I (1‐tigloyl‐3acetyl‐11‐ hydroxy‐ 11‐demethoxycarbonyl meliacarpin)	618.7	162.74	−8.41	−4.76	Low	No	Yes	1.81	0.17
Azadiradione	450.57	73.58	−5.63	−5.58	High	No	Yes	4.34	0.55
Deacetyl‐nimbin	498.56	112.27	−8.13	−3.72	High	No	No	2.76	0.55
Epoxyazadiradione	466.6	86.11	−6.14	−5.31	High	No	Yes	3.93	0.55
Nimbin	540.60	118.34	−7.98	−4.20	High	No	No	3.24	0.55
Nimbolin A	642.78	101.27	−5.31	−7.76	Low	No	Yes	5.83	0.17
Nimbandiol	456.5	106.20	−8.47	−3.06	High	No	Yes	2.31	0.55
Nimocinol	452.58	76.74	−5.70	−5.55	High	No	Yes	4.29	0.55
Nimbinene	482.6	92.04	−7.80	−3.83	High	No	No	3.44	0.55
Nimbocinone	470.68	66.76	−5.57	−5.69	High	No	No	4.94	0.55
Nimbocinolide	500.58	130.36	−7.75	−4.17	High	No	Yes	2.52	0.55
Nimbolide	466.52	92.04	−7.61	−3.94	High	No	Yes	3.11	0.55
Meliantriol	490.72	90.15	−5.74	−5.84	High	No	Yes	4.32	0.55
Caryophllene	204.35	0.00	−4.44	−3.87	Low	No	Yes	4.24	0.55
n‐hexacosanol	382.71	20.23	0.26	−8.52	Low	No	No	9.06	0.55
7‐desacetyl‐7‐ benzoyl‐azadiradione	528.64	86.11	−5.34	−6.78	High	No	Yes	4.95	0.55
17‐ hydroxyl‐azadiradione	466.57	93.81	−6.39	−5.09	High	No	Yes	3.66	0.55
Gedunin	482.57	95.34	−6.25	−5.40	High	No	Yes	3.73	0.55
Vepinin	452.58	65.74	−5.39	−5.82	High	No	Yes	4.38	0.55
Gallic acid	170.12	97.99	−6.84	−1.64	High	No	No	0.21	0.56

Abbreviations: BBB, blood brain barrier; BS, biostability; ESOL Log‐S, water solubility; GIA, gastrointestinal tract; Log‐Kp, Skin permeation; Log‐Po/w, lipophilicity consensus log; MW, molecular weight; P‐gp, P‐glycoprotein; TPSA, topological polar surface area.

### 3.8 Predicted Drug Targets

The accurate drug target interaction prediction plays a crucial role in drug discovery and repositioning, minimizing experimental expenses (Thafar et al. 2021). Most phytochemicals of *A. indica* bind to acetyl‐CoA carboxylase 2, mu opioid receptor, epoxide hydratase, and vitamin D receptors that trigger their pathways in dissimilar cascades. Binding probability is given in Table 7. Nimbolin A binds to cannabinoid receptor 2 and cyclin‐dependent kinase 2, caryophyllene targets glucocorticoid receptor and estrogen receptors alpha, and 7‐desacetyl‐7‐benzoylazadiradione skin permeable component binds specifically to kappa opioid receptor and vitamin D receptor.

**TABLE 7:** Predicted drug‐target interaction.

Phytochemicals	I.	II.	III.	IV.	V.	VI.	VII.	VIII.	IX.	X.	XI.	XII.	XIII.	XIV.
Azadiradione	40	40		10			40		10	10				
Nimbolin A	25	35	55	45	15				15				10	
Nimocinol	50			40			20	10		20			30	
Nimbocinone		45		55	40	35		30		20				20
Meliantriol			50	30		40	10	10		30				
Caryophllene								30			30	35		
7‐ desacetyl‐7‐ benzoylazadiradione		10	25	20		30	50	40	35	65				
Vepinin	10	40					20			55				

Abbreviations: I, Heat shock protein HSP‐90 alpha; II, Cathepsin S; III, Cannabinoid receptor 2; IV, Cyclin‐dependent kinase 2; IX, Cathepsin K; V, Acetyl‐ CoA Carboxylase 2; VI, Mu opioid receptor; VII, Epoxide hydratase; VIII, Vitamin D receptor; X, Kappa Opioid receptor; XI, Glucocorticoid Receptor; XII, Autotoxin; XIII, Estrogen receptor alpha; XIV, Insulin receptor; XV, Carbonic anhydrase IV.

### 3.9 Toxicity Evaluation of *A. indica* Phytochemicals

In order to chemically assess the risk, chemical toxicity prediction models and the admetSAR Web server provide insight into structural modeling (Rim 2020). The results of toxicity evaluation showed Nimocinol, Meliantriol, Caryophyllene, and Vepinin have metabolic sites as lysosomes while the rest of the phytochemicals metabolized in mitochondria by isozyme of Cytochrome P450. Biodegradation converts phytochemicals into non‐toxic substances. Aromatic chemicals' resistance increased by enzymes, resulting in lower fatal doses in fish and rats. As 7‐desacetyl‐7‐benzoylazadiradione shows 2.19 for rats and 1.9 for fish. Except for Nimbocinone and Meliantriol showing less doses as their LD as −3.9 and −2.7 for rat and fish models, respectively. Nimocinol, Caryophyllene, and Vepinin, are less irritant than others and have low potential for mutagenic, tumorigenic, or reproductive system effects (Table 8) indicating their safety for therapeutic and dermatocosmetic use.

**TABLE 8:** Data warrior and admetSAR predicted toxicity of phytochemicals.

Toxicity								
Phytochemicals	Subcellular localization	Biodegradation	Rat	Fish	Tumorigenic	Reproductive effective	Irritant	Mutagenic
Azadiradione	Mitochondria	Not readily biodegradable	1.24	0.5	None	None	None	None
Nimbolin A	Mitochondria	Not readily biodegradable	2.43	0.5	None	None	None	None
Nimocinol	Lysosome	Not readily biodegradable	2.56	1.3	None	None	Low	None
Nimbocinone	Mitochondria	Readily biodegradable	−3.9	0.6	None	None	None	None
Meliantriol	Lysosome	Readily biodegradable	1.36	−2.7	None	None	None	None
Caryophllene	Lysosome	Not readily biodegradable	−1.4	2.7	None	None	Low	None
7‐ desacetyl‐7‐ benzoylazadiradione	Mitochondria	Readily biodegradable	2.19	1.9	None	None	None	None
Vepinin	Lysosome	Not readily biodegradable	2.43	−2.7	None	None	Low	None

### 3.10 Homology Modeling

**FIGURE 3:**
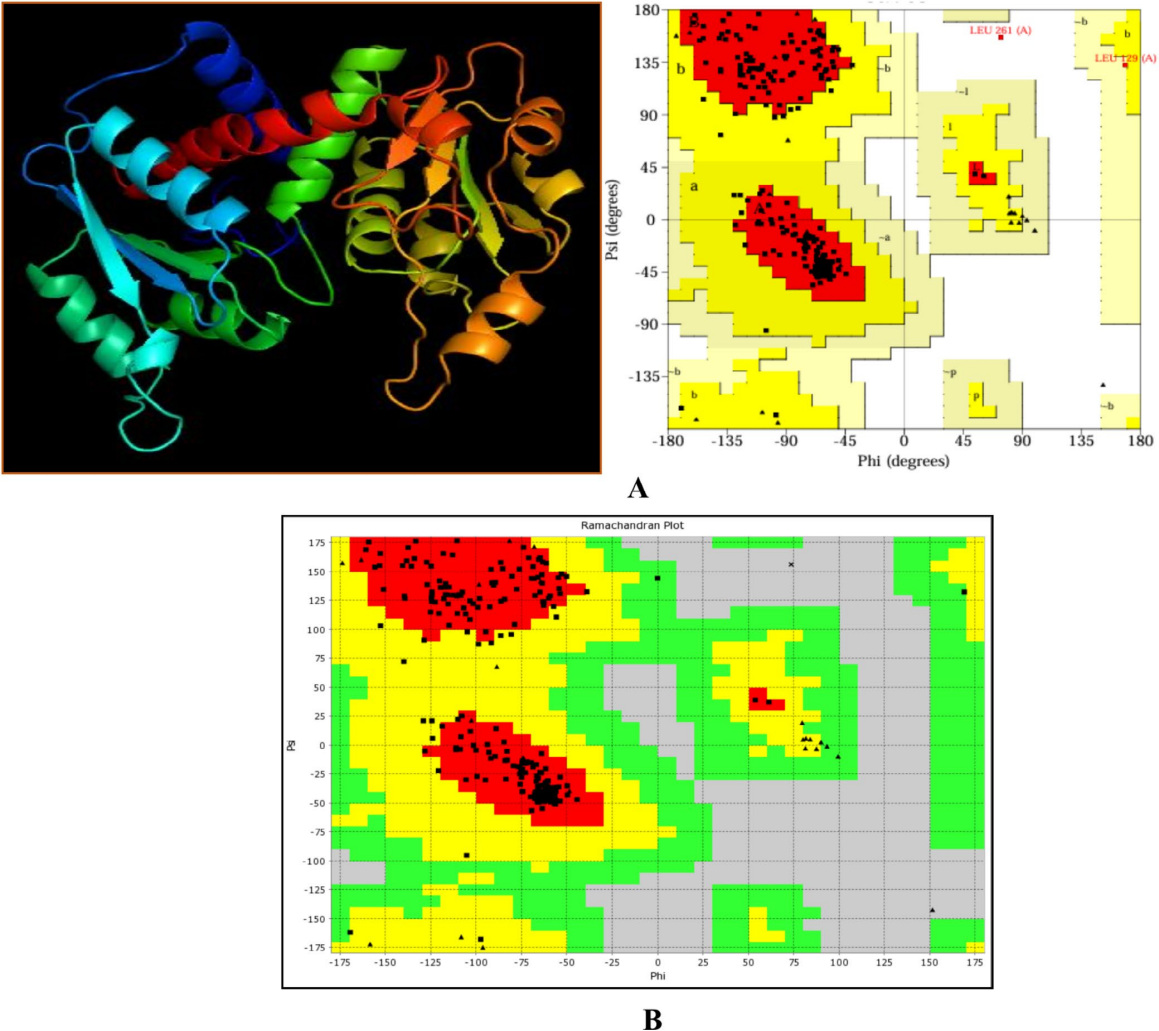
(A) Revealed structure of SsGST (B) Validation of SsGST by Ramachandran plot.

The Sequence identities of SsGST protein are 62.67% and coverages of 99% of the templates 4q5q.1 (Figure 3A). An SsGST protein was chosen and the model range was 219 amino acids. Reasonable structural similarity by root‐mean‐square deviation (RMSD) values of 0.067 for SsGST with its template. The 3‐dimensional protein model generates the Ramachandran plot for structure validation which revealed 92.5% of amino acids in the core region (Figure 3B). The forbidden region containing (0.4%), generously allotted region (0.4%) and additional region (6.7%) amino acids. Physicochemical properties of SsGST protein obtained from the ExPASy database are exhibited in Table 9.

**TABLE 9:** Physiochemical properties of SsGST.

Property	Value
Sequence length	219
Molecular weight	25,614
Charge	7.1
Isoelectric point	8.67
Aliphatic index	95.75
Molecular formula	C_1183_H_1824_N_298_O_323_S_7_
Total number of atoms	3635
Gravy	−0.27
Instability index	36.66
Estimated half‐life	The estimated half‐life is: 30 h (Mammalian reticulocytes, in vitro).
	> 20 h (yeast, in vivo).
	> 10 h ( *Escherichia coli* , in vivo).
A280 molar extinction coefficients	41,495 M^−1^/cm
Abs 0.1% (=1 g/L)	1.62

### 3.11 Molecular Docking

**FIGURE 4:**
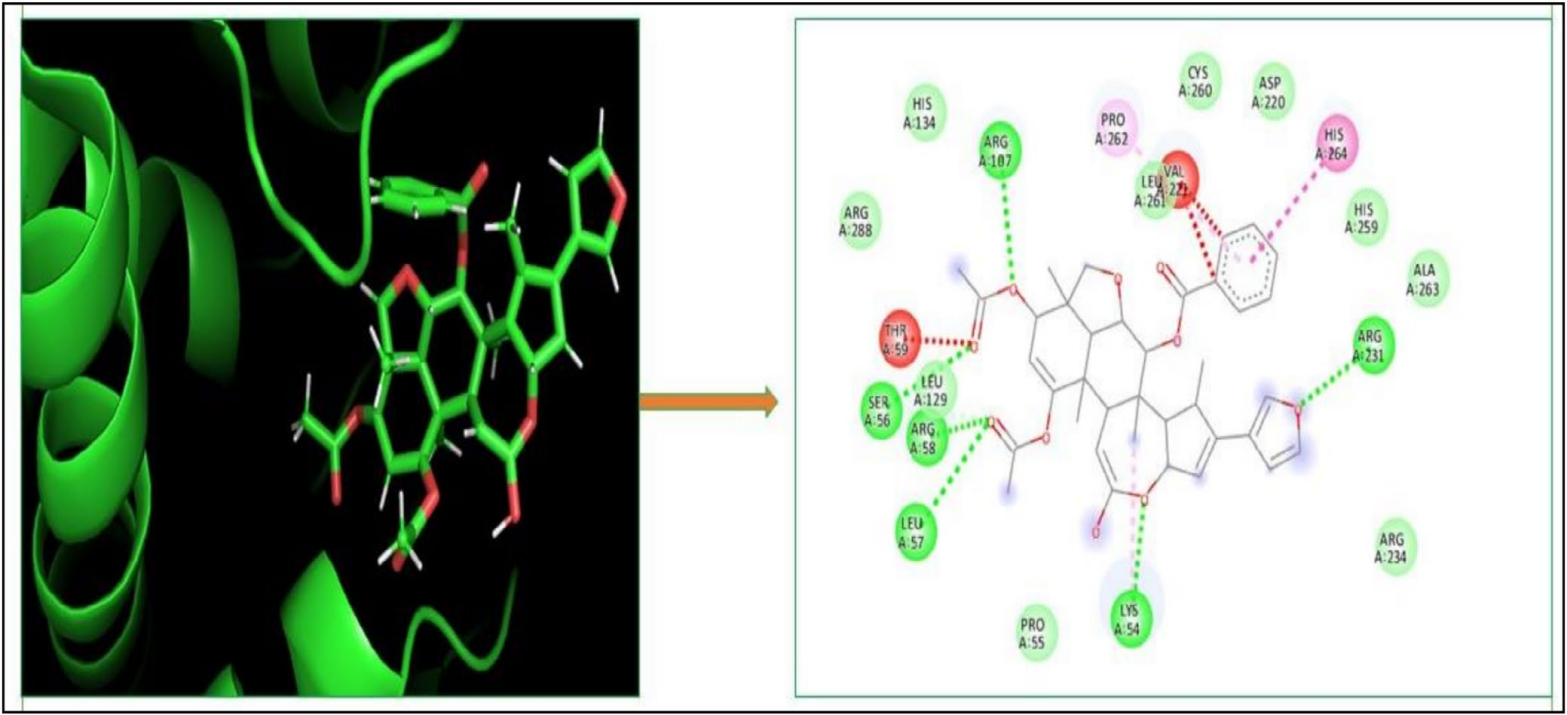
Receptor‐ligand interaction of docked phytochemical via Discovery studio.

The molecular docking provides insight into phenomena underlying the protein–ligand interaction (Chigurupati et al. 2022). Phytochemicals of *A. indica* demonstrate potential inhibition against the targeted SsGST protein (Figure 4). The most promising result was shown by 7‐desacetyl‐7‐benzoylazadiradione, having a binding score of −12.324 kcal/mol, followed by Vepinin (−11.567 kcal/mol) and Nimbocinone (−11.451 kcal/mol), while Azadiradione shows no binding capacity with the SsGST protein, as given in Table 10. The 2D structure of 7‐desacetyl‐7‐benzoylazadiradione demonstrates amino acids ARG: 58, SER: 56, LEU: 57, LYS: 54, and ARG: 107 participate in forming hydrogen bonds. THR: 59 contributes an unfavorable bump in the receptor‐ligand complex. Carbon‐mediated hydrogen bonds are generated by PRO: 55 and HIS: 134 amino acids (File S2). These findings suggest that if this phytochemical is isolated from *A. indica* , it would serve as a viable alternative in the treatment of scabies.

**TABLE 10:** Binding scores of phytochemicals to SsGST.

Name	Binding affinity (kcal/mol)	Interacting residues
Azadiradione	Error of Biding Pose	—
Nimbolin A	−8.321	GLN A:79, LEU A:97, ASN A:96
Nimocinol	Error of Biding Pose	—
Nimbocinone	−11.451	ASP A: 220, ARG A:107, LEU A:129
Meliantriol	−10.324	THR A:223, VAL A:221, LEU A:261
Caryophllene	−6.312	ALA A:49, PHE A:100, LEU A:77
7‐ desacetyl‐7‐ benzoylazadiradione	−12.324	ARG: 58, SER: 56, LEU: 57

### 3.12 Network Gene Expression

**FIGURE 5:**
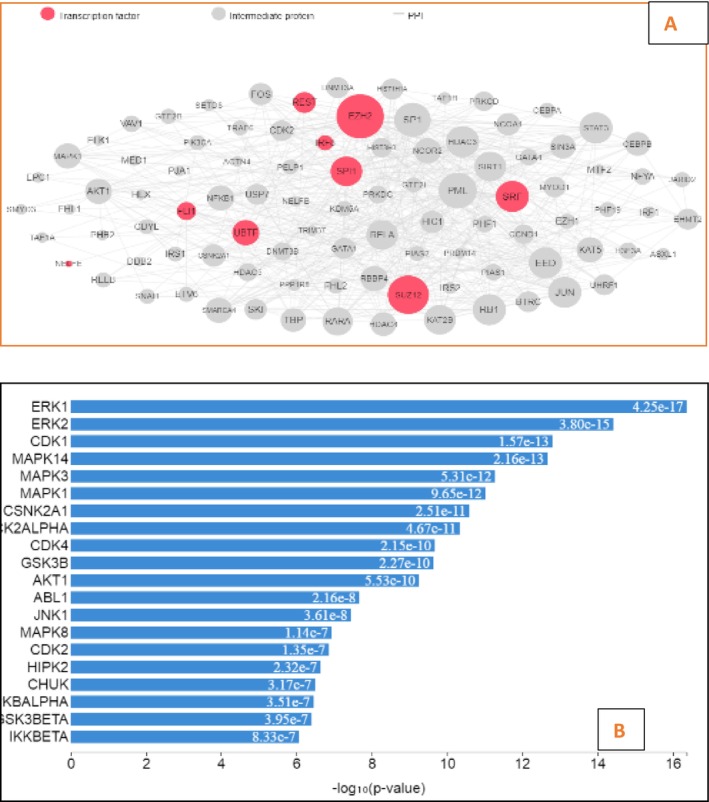
(A) Expression2Kinases network (B) Kinase enrichment analysis.

This analysis substantiates the probable pathway by which phytoconstituents show acaricidal activity (Fantatto et al. 2022). All of the target gene IDs with the highest probability to 7‐ desacetyl‐7‐ benzoylazadiradione were utilized. The showed interactions of transcription factors, kinases, and intermediate proteins are depicted in Figure 5, which has a high hyper geometric (−log10) *p* value. The transcription factors include CTSV, MAPK11, FASN, WNT3A, GSK3B, AKT1, ABL1, IKKBETA, CSNK2A1, SRF, SUZ12, UBTF, EZH2, and REST.

## 4 Conclusion

This study provides an exhaustive assessment of *Azadirachta indica's* acaricidal, antibacterial, antioxidant, and enzyme inhibitory potential against *Sarcoptes scabiei*. Through an integrated in vitro and in silico approach, the bioactive compound of *A. indica* , particularly 7‐desacetyl‐7‐benzoylazadiradione, has demonstrated significant inhibitory effects on glutathione S‐transferase (SsGST), a critical enzyme in mite detoxification. The in vitro assay confirmed its dose‐dependent acaricidal efficacy, yielding substantial mite mortality. The extract exhibited broad‐spectrum antimicrobial activity with a notable inhibition zone against *Escherichia coli* , *Bacillus subtilis, Clostridium perfringens* and *Streptococcus equinus*. It also exhibited potent antioxidant properties, with an IC_50_ of 3.15 mg/mL, underscoring its role in alleviating oxidative stress. The safety of *A. indica* was further confirmed by hemolytic activity. Enzyme inhibition assays demonstrated significant modulation of acetylcholinesterase (AChE) and glutathione S‐transferase (GST), suggesting potential neuroactivity and disruption of detoxification pathways. These results are corroborated by in silico analysis highlighting its skin permeability, low toxicity, probable pathway and strong binding affinity against SsGST active site residues. Further in vivo validation, drug development, and clinical trials would pave the way towards its practical application.

## Author Contributions

**Tehreem Fatima:** data curation (equal), formal analysis (equal), investigation (lead), methodology (lead), software (equal), writing – original draft (lead). **Mazhar Abbas:** conceptualization (lead), formal analysis (equal), project administration (lead), resources (equal), software (equal), supervision (equal), validation (equal), visualization (equal). **Kinza Zafar:** formal analysis (equal), investigation (equal), visualization (equal), writing – original draft (equal). **Maha Gul Zafar:** formal analysis (equal), investigation (equal), validation (equal), writing – review and editing (equal). **Waqas Haider:** formal analysis (equal), investigation (equal), validation (equal), writing – review and editing (equal). **Muhammad Haseeb Zafar:** validation (equal), visualization (equal), writing – review and editing (equal). **Muhammad Riaz:** formal analysis (equal), validation (equal), visualization (equal), writing – original draft (equal). **Munawar Iqbal:** formal analysis (equal), validation (equal), visualization (equal), writing – review and editing (equal). **Andrew G. Mtewa:** formal analysis (equal), validation (equal), visualization (equal), writing – review and editing (equal).

## Ethics Statement

The authors have nothing to report.

## Conflicts of Interest

The authors declare no conflicts of interest.

## Supporting information


**Data S1:** fsn371204‐sup‐0001‐DataS1.docx

**Data S2:** fsn371204‐sup‐0002‐DataS2.xlsx

## Data Availability

Data will be available from principal and corresponding authors on reasonable request.
