# Supplementary material for: Unlocking the Antioxidant, Enzyme Inhibitory and Acaricidal Potential of Azadirachta indica Phytoconstituents Using In Vitro and In Silico Approaches
Source: Food Sci Nutr. 2025 Nov 29;13(12):e71204. doi: 10.1002/fsn3.71204 (PMC12664285; doi:10.1002/fsn3.71204)

**2D structure interaction of *A. indica* derived phytochemicals against SsGST**

**1.Nimbolin A 2. Nimbocinone**


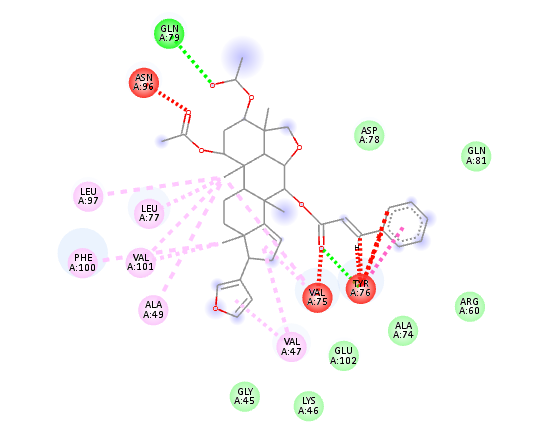

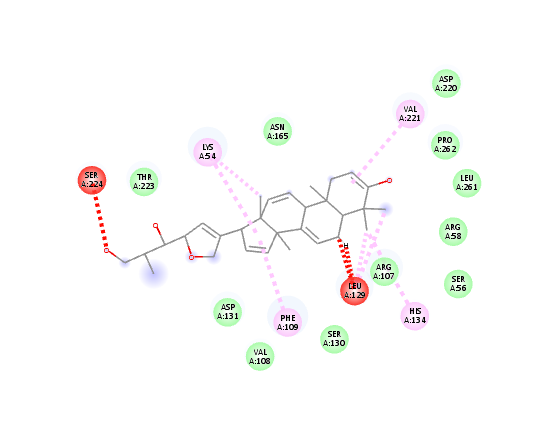


**3. Melintriol 4. Caryophllene**


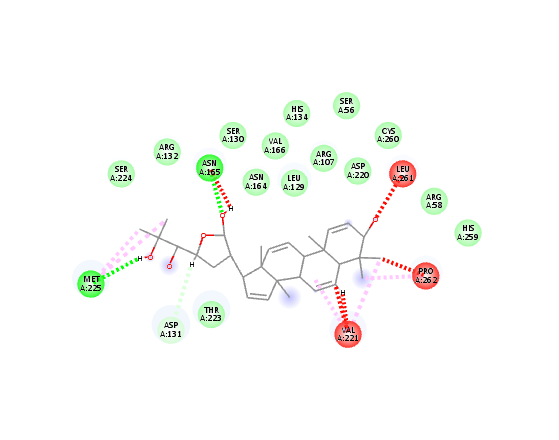

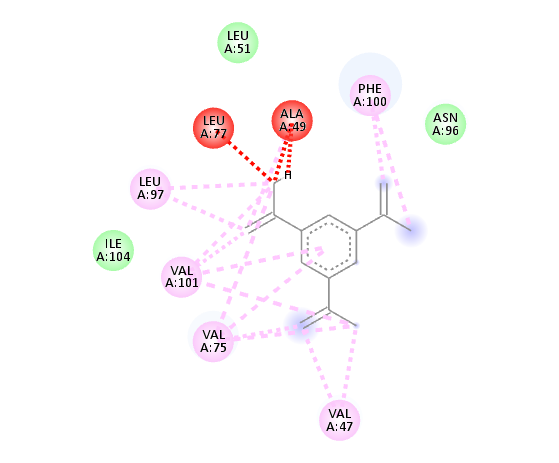


**5. 7- desacetyl-7- benzoylazadiradione**


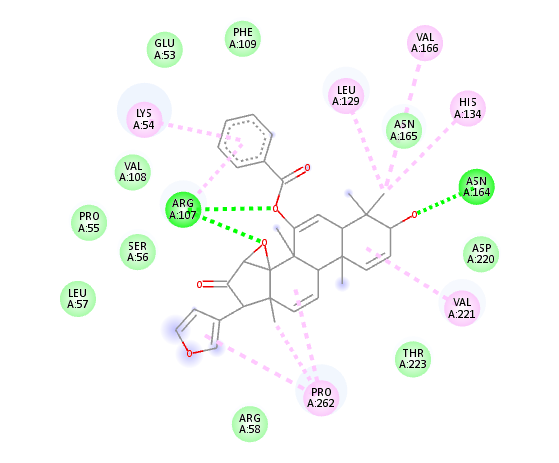

Supplement: Supplementary file 1 — Data S1: fsn371204‐sup‐0001‐DataS1.docx [file FSN3-13-e71204-s002.docx]
